# Barriers and facilitators to care for individuals with sickle cell disease in central North Carolina: The emergency department providers’ perspective

**DOI:** 10.1371/journal.pone.0216414

**Published:** 2019-05-07

**Authors:** Rita Vanessa Masese, Dominique Bulgin, Christian Douglas, Nirmish Shah, Paula Tanabe

**Affiliations:** 1 Duke University School of Nursing, Durham, North Carolina, United States of America; 2 Duke University Office of Clinical Research, Durham, North Carolina, United States of America; 3 Duke University Medical Center, Durham, North Carolina, United States of America; Medical University Graz, AUSTRIA

## Abstract

**Background:**

Sickle cell disease (SCD) is an inherited blood disorder associated with acute pain crisis and other complications that lead to frequent emergency department (ED) visits. To improve outcomes, the National Heart, Lung and Blood Institute (NHLBI) published recommendations for management of acute pain crisis. NHLBI also funded eight centers across the United States to participate in the Sickle Cell Disease Implementation Consortium. This six-year effort consists of two phases. Phase one involved conducting needs assessments of barriers and facilitators to SCD care. The aim of this study was to describe challenges and facilitators to caring for SCD from the perspective of ED providers in central North Carolina (NC).

**Methods and findings:**

We conducted a needs assessment survey with ED providers throughout NC. We also conducted focus groups and an interview with ED providers from three healthcare facilities in central NC. One hundred and eleven surveys (60.6% physicians, 26% registered nurses, 13.5% physician assistants) were completed and 13 providers participated in 3 focus groups and 1 interview. Slightly more than half (50. 4%) utilized individualized dosing protocols to treat sickle cell pain. Only 32.4% of the providers were aware of the NHLBI SCD recommendations. Barriers to care from the survey included: patient behavior (67.57%), the opioid epidemic (67.57%), overcrowding (64.86%), and concern about addiction (49.55%). Perceived barriers to care identified in the focus groups and interview included: high patient volumes, lack of SCD care protocols, poor communication among providers and stigma. Facilitators to care included: individualized pain plans, comfort prescribing opioids and electronic medical records.

**Conclusion:**

SCD care is influenced by many factors. Our results illuminate the need for increased use of the NHLBI SCD recommendations, individualized pain protocols, and use of electronic medical records and other care-interventions, specifically geared towards improving provider knowledge and mitigating provider bias.

## Introduction

Sickle cell disease (SCD) is an inherited blood disorder with a predominant prevalence among the African-American population in the United States and is associated with several co-morbidities, including stroke, renal failure, and sepsis [1]. The median lifespan is 42 and 48 years among males and females respectively [2]. Individuals also experience both acute and chronic pain conditions. The complexity of sickle cell disease (SCD) ultimately results in frequent hospital admissions and emergency department (ED) visits.

In 2006 there were over 200,000 emergency department (ED) visits for sickle cell disease (SCD); pain is noted to be the most common reason for emergency department (ED) visits [3]. One of every 4 ED visits by an individual with SCD led to a hospital admission, resulting in a high associated cost [4]. In 2006 the estimated total of 232,382 ED visits and 68,410 hospitalizations from the ED, accounted for an estimated $2.4 billion dollars [3]. When compared to congestive heart failure during 2006, the number of hospital admissions per 100 persons with SCD was significantly higher for SCD (68.4 vs. 17.3) as well as the charges per 100 patients ($1.5 million for SCD, vs. $500,000 for congestive heart failure) [3]. In 2010, SCD was associated with the highest 30-day re-admission rate among all diagnoses, 31.9% [5].

In an attempt to provide guidance to SCD healthcare providers, in 2014 the National Heart, Lung and Blood Institute (NHLBI) published recommendations for the treatment of SCD [1]. The document included 17 recommendations for treatment of painful vaso-occlusive episodes. Specific recommendations included assigning a triage score of level 2, administration of opioids within 60 minutes of arrival, use of parenteral opioids, and use of individualized analgesic dosing protocols whenever possible. However, there are barriers to the implementation of these guidelines leading to frustration with the ED by both patients and healthcare providers [6–8].

In an effort to improve outcomes for persons with SCD, the National Heart, Lung and Blood Institute (NHLBI) funded eight centers across the US to participate in the Sickle Cell Disease Implementation Consortium (SCDIC, U01HL133964). This six-year effort consists of two phases. During Phase 1, each site conducted a needs assessment of barriers to care from the patients’, primary care providers’, SCD specialists’, and ED providers’ perspectives. In this paper, we report the results of the needs assessment (surveys and focus groups) of facilitators and barriers to care for patients with SCD in North Carolina, from the perspective of emergency medicine providers.

The purpose of the needs assessment was to develop a more complete understanding of facilitators and barriers to care for individuals with SCD from the perspective of ED providers, as well as to assess current adherence and knowledge of the National Heart, Lung and Blood Institute (NHLBI) recommendations for treating vaso-occlusive episodes. This data will be used to develop interventions for phase two, which aims to improve ED management of SCD and adherence to the NHLBI recommendations for the treatment of vaso-occlusive episodes.

## Methods

### Study design

A combination of needs assessment surveys, focus groups, and an individual interview were used. Institutional review board approval from Duke University was obtained prior to data collection (Protocol ID: Pro00073506). Participation in the study was voluntary and participants could withdraw from the study at any time. A waiver of written consent was provided for survey completion. Focus group and interview participants signed an informed written consent. Personal identifying information was not collected in order to preserve participant confidentiality.

### Study sample and setting

Eligibility criteria included: 1) licensure as a physician, registered nurse or physician assistant, 2) current employment in an ED, and 3) provision of care to persons with SCD. Survey participants were recruited via emails sent from their hospital administrators. To encourage generalizability of the responses, the study team worked with the North Carolina Emergency Nurses Association. An electronic link to the survey was displayed on their website. The organization also sent the link to the email list serve for their members, thus resulting in responses from across North Carolina.

Focus group and interview participants were purposefully recruited from three hospitals with high volumes of SCD patients in North Carolina. To increase generalizability, two of the hospital sites were associated with large comprehensive SCD care centers, the other site was not. For the purposes of this study, the site not associated with a comprehensive SCD care center was referred to as hospital A; the other two sites were referred to as hospital B and C. Providers interested in the study contacted the study team to schedule participation. Interview participants were invited to complete the needs assessment survey at the time of the focus group or individual interview.

### Data collection

#### Surveys

The needs assessment survey questions were developed by members of the Sickle Cell Disease Implementation Consortium’s needs assessment committee. The committee included at least one member from the 8 participating sites with leadership from the Research Triangle Institute, the data coordinating center for the Sickle Cell Disease Implementation Consortium. The survey was divided into four sections: 1) demographic and practice characteristics, 2) facilitators to treatment of SCD (n = 13 items), 3) barriers to treatment, (14 items and “other), and 4) questions about ED vaso-occlusive episode treatment and opioid prescribing practices (n = 4). Each facilitator item was endorsed using a 4 point Likert scale (strongly disagree, disagree, agree or strongly agree). Responses were re-coded for analysis (strongly disagree/disagree or agree/strongly agree). The overall mean (SD) was also calculated per item. Participants selected all barriers that were relevant to their practice and answered each of the items about ED vaso-occlusive episode treatment and prescribing practices (use of ED vaso-occlusive episode protocols, use of individualized vaso-occlusive episode protocols, awareness of NHLBI recommendations and whether or not they prescribe Schedule II opioids for SCD patients at discharge). The survey contained novel questions, validated instruments and items from previous literature on barriers to SCD. Personal identifying information was not collected in order to preserve participant confidentiality and encourage responses. The survey was administered electronically from June to September 2017.

#### Interviews

Focus groups were conducted from August to October 2017. Each session lasted approximately 90 minutes and was led by the principal and co-investigators who have extensive training in qualitative research methods. The study utilized a semi-structured interview guide with broad opening questions and more specific follow-up probes. The questions were developed by the same members of the Sickle Cell Disease Implementation Consortium’s needs assessment group that developed the surveys. The guide explored barriers and facilitators to SCD management, reasons for SCD patient ED visit, knowledge of SCD management, comfort with opioid prescriptions and SCD co-management. Data collection continued until data saturation, where no new insights could be obtained [9]. Interviews were audio-recorded and transcribed verbatim. Interview participants were compensated $30. There was no compensation for completing the needs assessment surveys.

#### Data analysis

Quantitative data from the needs assessment survey were entered into a Research Electronic Data Capture (REDCap) database. SAS statistical software (version 9.4) was used to conduct descriptive analyses.

QSR NVivo 11^TM^ software was used to code, organize and manage qualitative data. Codes were developed deductively from topics in the interview guide and inductively from the content in the transcripts. Codes were compiled into a codebook with definitions, inclusion and exclusion criteria. The codebook was reviewed by the research team for agreement before being applied to the transcripts. Two members of the research team independently coded portions of the transcripts, discussed and compared coding decisions. Disagreements were resolved through repeated discussions until consensus was achieved. Agreement ranged from 92% to 100% with a weighted kappa value of 0.98. After completion of coding, the coded texts were arranged into categories and subcategories based on codes that related to each other. The salience of particular codes was determined by the frequency of application, similarities and differences among study participants.

## Results

### Participant demographic data

The needs assessment survey was completed by 111 ED providers (demographic and practice characteristics are summarized in Table 1). Majority of participants who reported practicing in an urban setting were medical doctors. 13 ED providers from the 3 different care settings in North Carolina participated in 3 focus groups and 1 interview. Participants reported providing care to approximately 1 to 2 SCD patients per week who were mostly adults. The most common reported reason for SCD patient visit was pain crisis. Other reasons for presentation included stroke, acute chest syndrome, priapism, splenic sequestration, and fever.

**Table 1:** Demographic and practice characteristics (surveys, focus groups and interview).

Characteristics	Surveys (n = 111)	Focus Groups/Interview (n = 13)
**Age, mean (sd)**	37.66 (8.95)	32.67 (14.73)
Missing, n	34	0
**Sex, n (%)**		
Female	52 (52.0)	9 (69.2)
Male	48 (48.0)	4 (30.8)
Missing, n	11	
**Race, n (%)**		
White	86 (89.6)	9 (69.2)
Black/African American	3 (3.1)	1 (7.7)
Asian	6 (6.3)	2 (15.4)
Other	1 (1.0)^a^	1 (7.7)^b^
Missing, n	15	
**Years of Practice, mean (sd)**	10.75 (8.01)	11.54 (13.42)
Missing, n	10	1
**Specialty type, n (%)**		
MD	63 (60.6)	6 (50.0)
PA	14 (13.5)	2 (16.7)
RN	27 (26.0)	4 (33.3)
Missing, n	7	1
**Urban practice setting, n (%)**		
Rural	9 (8.7)	0 (0.0)
Urban	67 (65.0)	10 (83.3)
Suburban	27 (26.2)	2 (16.7)
Missing, n	8	1

Missing, participants who provided no response to the survey item; N, total number.

^a^1 provider identified as Other (Pacific Islander).

^b^ 1 provider identified as Other (multiple races).

### Survey findings

**Fig 1:**
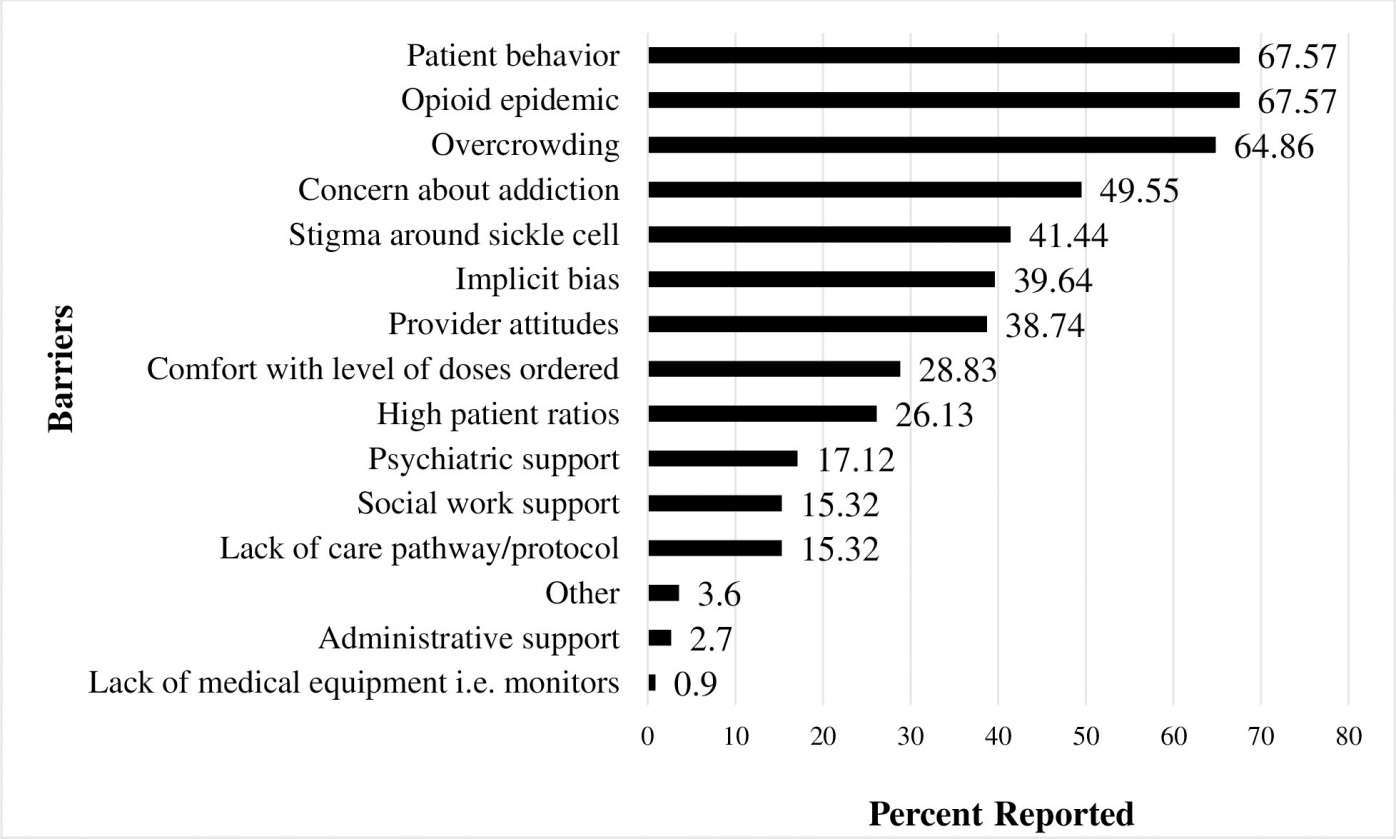
Survey identified barriers to care in the emergency department.

Table 2 presents survey responses. In general, participants reported good infrastructure and support to provide care to persons with SCD with the exception of workflow. Participants also reported difficulty making referrals to both SCD specialists and primary care providers. Fig 1 reports the percentages of identified barriers to care. The most frequent barriers were patient behavior (67.57%), the opioid epidemic (67.57%) and ED overcrowding (64.86%). Table 3 reports findings related to use of NHLBI recommendations. A majority (66.7%) of ED providers reported not being aware of the NHLBI recommendations for the treatment of SCD.

**Table 2:** Survey results on factors influencing care provision in the emergency department.

Barriers to care	Disagree/ Strongly Disagree N (%)	Agree/ Strongly Agree N (%)	Mean (sd)	Missing, n
**I have the knowledge to provide care to persons with SCD.**	**2 (1.8)**	**109 (98.2)**	**3.5 (0.54)**	**0**
MD	1 (1.6)	62 (98.4)	3.6 (0.52)	0
PA	0 (0.0)	14 (100.0)	3.4 (0.50)	0
RN	0 (0.0)	27 (100.0)	3.4 (0.50)	0
Missing	1 (14.3)	6 (85.7)	3.0 (0.58)	0
**I have the training to deliver care to the person with SCD.**	**4 (3.6)**	**106 (96.4)**	**3.5 (0.57)**	**1**
MD	1 (1.6)	62 (98.4)	3.6 (0.51)	0
PA	0 (0.0)	14 (100.0)	3.4 (0.51)	0
RN	1 (3.8)	25 (96.2)	3.4 (0.57)	1
Missing	2 (28.6)	5 (71.4)	3.0 (0.82)	0
**I have the administrative support I need to treat patients with SCD.**	**2 (2.0)**	**100 (98.0)**	**3.5 (0.57)**	**9**
MD	0 (0.0)	58 (100.0)	3.6 (0.50)	5
PA	0 (0.0)	14 (100.0)	3.4 (0.51)	0
RN	2 (8.3)	22 991.7)	3.2 (0.74)	3
Missing	0 (0.0)	6 (100.0)	3.3 (0.52)	1
**I have access to medications I need to treat pain in individuals with SCD.**	**2 (1.8)**	**106 (98.1)**	**3.6 (0.53)**	**3**
MD	0 (0.0)	60 (100.0)	3.7 (0.47)	3
PA	0 (0.0)	14 (100.0)	3.4 (0.51)	0
RN	2 (7.4)	25 (92.6)	3.4 (0.64)	0
Missing	0 (0.0)	7 (100.0)	3.3 (0.49)	0
**I am able to make a follow-up appointment with a sickle cell specialist following discharge.**	**58 (59.8)**	**39 (40.2)**	**2.3 (0.83)**	**14**
MD	31 (51.7)	29 (48.3)	2.5 (0.81)	3
PA	7 (58.3)	5 (41.7)	2.3 (0.89)	2
RN	17 (80.9)	4 (19.1)	2.0 (0.74)	6
Missing	3 (75.0)	1 (25.0)	1.8 (0.96)	3
**I am able to make a follow-up appointment with a primary care provider following discharge.**	**57 (57.0)**	**43 (43.0)**	**2.4 (0.84)**	**11**
MD	32 (51.6)	30 (48.4)	2.5 (0.80)	1
PA	6 (50.0)	6 (50.0)	2.5 (0.80)	2
RN	16 (76.2)	5 (23.8)	2.0 (0.80)	6
Missing	3 (60.0)	2 (40.0)	2.2 (1.30)	2
**I am able to refer patients to a case management program upon discharge.**	**32 (33.3)**	**64 (66.7)**	**2.8 (0.89)**	**15**
MD	14 (24.1)	44 (75.9)	3.0 (0.85)	5
PA	4 (30.8)	9 (69.2)	2.7 (1.11)	1
RN	12 (60.0)	8 (40.0)	2.2 (0.72)	7
Missing	2 (40.)	3 (60.0)	2.6 (0.55)	2
**I work in an ED with sufficient nurse staffing to provide good pain management to persons with SCD**.	**20 (18.2)**	**90 (81.8)**	**3.1 (0.69)**	**1**
MD	8 (12.9)	54 (87.1)	3.2 (0.70)	1
PA	2 (14.3)	12 (85.7)	3.1 (0.62)	0
RN	9 (33.3)	18 (66.7)	2.8 (0.72)	0
Missing	1 (14.3)	6 (85.7)	2.9 (0.38)	0
**I work in an ED with sufficient physician/provider staffing to provide good pain management to persons with SCD.**	**10 (9.1)**	**100 (90.9)**	**3.2 (0.63)**	**1**
MD	2 (3.2)	61 (96.8)	3.4 (0.55)	0
PA	0 (0.0)	13 (100.0)	3.3 (0.48)	1
RN	7 (25.9)	20 (74.1)	2.9 (0.78)	0
Missing	1 (14.3)	6 (85.7)	2.9 (0.38)	0
**Nursing staff ratios allow our ED to provide safe care.**	**21 (19.4)**	**87 (80.6)**	**3.0 (0.68)**	**3**
MD	10 (16.4)	51 (83.6)	3.1 (0.72)	2
PA	2 (15.4)	11 (84.6)	2.9 (0.49)	1
RN	8 (29.6)	19 (70.4)	2.9 (0.70)	0
Missing	1 (14.3)	6 (85.7)	2.9 (0.38)	0
**Our nursing staffing allows our ED to provide high-quality care.**	**20 (18.2)**	**90 (81.8)**	**3.1 (0.73)**	**1**
MD	8 (12.9)	54 (87.1)	3.3 (0.73)	1
PA	3 (21.4)	11 (78.6)	2.9 (0.62)	0
RN	8 (29.7))	19 (70.4)	2.9 (0.73)	0
Missing	1 (14.3)	6 (85.7)	2.7 (0.76)	0
**A lack of insurance, or being under insured does not affect my ability to provide good care**.	**10 (9.3)**	**97 (90.7)**	**3.4 (0.72)**	**4**
MD	9 (15.0)	51 (85.0)	3.4 (0.82)	3
PA	0 (0.0)	14 (100.0)	3.7 (0.47)	0
RN	1 (3.7)	26 (96.3)	3.4 (0.58)	0
Missing	0 (0.0)	6 (100.0)	3.5 (0.55)	1
**The workflow in our ED is conducive to proving high quality care for sickle cell pain crises.**	**33 (30.8)**	**74 (69.2)**	**2.8 (0.75)**	**4**
MD	15 (25.0)	45 (75.0)	2.9 (0.77)	3
PA	5 (35.7)	9 (64.3)	2.7 (0.61)	0
RN	11 (40.7)	16 (59.3)	2.7 (0.81)	0
Missing	2 (33.3)	4 (66.7)	2.7 (0.52)	1

Missing, participants who provided no response to the survey item; N, total number.

**Table 3:** Adherence to NHLBI recommendations and opioid prescribing patterns.

Items	N (%)
**Does your ED have a protocol for treating sickle cell pain?**	
Yes	77 (69.4)
No	24 (21.6)
Don’t know	10 (9.0)
**Does your ED use individualized dosing protocols to treat sickle cell pain?**	
Yes	56 (50.4)
No	36 (32.4)
Don’t know	18 (16.2)
Prefer not to answer	1 (0.9)
**Are you aware of the NHLBI recommendations for the treatment of VOC?**	
Yes	36 (32.4)
No	74 (66.7)
Prefer not to respond	1 (0.9)
**Upon discharge from the ED for sickle cell pain, do you prescribe Scheduled-II medications (i.e. opioid analgesics) to patients who request them?**	
Yes	38 (34.9)
No	60 (55.0)
I have never taken care of a SCD patient	2 (1.8)
I have never been asked by an SCD patient for opioid analgesics for their pain management	6 (5.5)
Prefer not to respond	3 (2.7)
Missing, n	2

Missing, participants who provided no response to the survey item; N, total number.

NHLBI, National Heart, Lung and Blood Institute

### Focus group and interview findings

Analysis of qualitative data from the three healthcare facilities (Hospital A, B and C) revealed similar and overlapping themes concerning barriers and facilitators to care of persons with SCD in the ED. Participants also made several recommendations to improve care.

#### Barriers to care

Barriers to care reported during the interviews included: 1) ED patient volume; 2) lack of SCD care protocols; 3) poor communication among providers; and 4) stigma and bias.

High patient volume and overcrowding of the ED was identified as a significant barrier to care at all the sites. Participants reported that due to the high volumes they tended to focus more on patients with high triage levels, limiting their ability to address and assess SCD patients in a timely manner. Overcrowding in the ED was compounded by constraints such as time, inadequate staffing, or lack of resources to treat SCD pain.

“E*D volume as a whole*, *and when we have fifty people in the waiting room as we often do*, *although I put the order in for every 20 minutes as needed*, *I’m sure that it’s often the actual amount of time it takes for them to get that medicine is perhaps even double*. *The same in the pods*. *Our nurses generally will have 4 patients each and if one of their patients is very ill then you can imagine they’re not able to get to that patient with sickle cell’s bedside as quickly as of course we would want to*, *to address their pain and assess it more frequently as we should*.” (Participant 7, Hospital B, MD)

Participants had varied responses about the types of pain management and analgesic they offered. Variation in pain management was attributed to a lack of standardized protocols and individualized pain plans in the ED. The healthcare system associated with Hospital A had a “Dilaudid free” (Hydromorphone free) policy that acted as a barrier to care by limiting or delaying access to pain medications. One participant remarked, “*I mean some of the Dilaudid free EDs it just it ends up*, *I think you can still prescribe it but it’s just very difficult and like the nurse has to get special approval*. *But if you’re in*, *you know*, *someone’s demanding Dilaudid and they’re in one of our facilities that has a Dilaudid free ED then we’re just delaying their treatment of pain*.” (Participant 1, Hospital A, MD)

Participants used a multidisciplinary approach to managing SCD patients, including relying on clinical notes and care plans crafted by the patient’s hematologists or pain specialists. Participants reported reaching out to the specialist if patients were not responding to standard therapy. However, most of the patients presented over the weekends and at night making it difficult for the participants to contact their hematologist or primary care physician. Inadequate communication with primary care providers also limited the participants’ ability to establish follow-up appointments for their patients. Lack of follow-up appointments with primary care physicians and hematologists perpetuated a cycle of patients receiving their care primarily in the ED.

Participants expressed concerns that patients may be drug seeking, and reported that that perception influenced the care they provided. They were aware of how stigmatizing thoughts impacted the care they provided. Participants from Hospital A used language commonly associated with bias and negative attitudes, such as “frequent flyers’ and “sickler.” One participant mentioned a discrepancy in the treatment of SCD pain compared to pain caused by other illnesses, revealing that providers may be more willing to offer opioids to patients with cancer or other co-morbidities than to patients with SCD.

“I*’ve had patients where you know I’m like I’m going to go see such and such which may be known to most of the staff that’s in the ER and I’ve heard the commentary of I just can’t stand them or you know just something derogatory*. *There’s one patient in particular I can think of who’s actually extremely polite but does present very*, *very*, *very often and I can just recall a few people telling me just how much they’re aggravated by that patient and I think people forget*, *just as nurses*, *that we have to examine ourselves and regulate our own biases before we go into the rooms because it’s not really my position to judge but I think that’s often forgotten*.” (Participant 10, Hospital C, RN)

#### Facilitators to care

Reported facilitators to providing care included: 1) individualized pain plans 2) comfort prescribing opioids, and 3) electronic medical records.

Individualized care and pain plans drafted by the patients’ hematologist or primary care provider with instructions for nurses on what opioid dosages to administer to the patient facilitated care delivery in the ED. Participants were able to determine if their patients were stable enough for discharge or required hospital admission based on their patients’ response to their care plan. Individualized care plans also mitigated biases in instances where patients requested for high dosages of opioids since the patient’s request could be confirmed by their individualized care plan.

“I*t sets expectations too*. *I think if having patient expectations saying like I know this is my care plan*, *I know that I’m not going to get an eyebrow raised if I ask for 3 milligrams of Dilaudid because it’s in my care plan that 3 milligrams of Dilaudid is my dose*. *So I think that setting expectations with patients through care plans is extra helpful*.” (Participant 3 Hospital A, PA)

Participants reported comfort prescribing and administering opioids as a facilitator to care. Comfort prescribing opioids was attributed to prior experience treating SCD patients and if there was a consistent record of usage of high doses of opioids in prior ED visits.

All participants reported that electronic medical records equipped them with vital knowledge about their patients’ history, including care plans, recent visits and medications lists. Information in electronic medical records also enabled providers to assess patient adherence to treatment and medical appointments.

#### Recommendations to improve care

Recommendations to improve care of SCD patients included: 1) comprehensive SCD care; 2) standardized care; 3) increased knowledge and empathy; and 4) community resources.

Participants, particularly those who worked in a non-comprehensive SCD center (Hospital A), reported that more centralized comprehensive SCD care could improve SCD patient outcomes.

Participants advocated for the development of standardized protocols for pain management. They urged that a protocol would ensure consistency of care, especially in ED settings that are learning institutions with high rates of clinician turnover.

Several participants recommended that care of SCD could be improved by more knowledge and understanding of the disease. They reported that improved knowledge of the disease could increase empathy and mitigate stigma and bias towards patients.

*“*I *think honestly just understanding and just the nature of the disease I mean we're all aware of what it is*, *what it causes and everything but kind of like I said*, *most of us don't have sickle cell so we don't fully grasp…*.*”* (Participant 13, Hospital B, ED Nurse)

ED providers thought that SCD patients could benefit from additional resources, including social support and alternative pain management. Participants also recommended the involvement of psychologist and social workers.

*“*I *would say you could make a huge difference with a very small frequent flier population by having almost like multiple resources available to them so a lot of times they’re not necessarily coming in just for sickle cell pain crisis*. *They have social issues*, *they have you know family issues*, *they have psychiatric issues*, *they have substance abuse issues*. *I think it’s a small percentage of the overall local sickle cell population that we’re seeing over and over and over and over again so I think if you could focus some kind of multidisciplinary efforts on those patients*, *psychiatry*, *social work*, *physical therapy*, *substance abuse counseling; if you could get all that together in a local spot and plug these people in I think you could probably make a big difference*.*”* (Participant 5, Hospital A, MD)

## Discussion

We conducted focus groups and surveys with ED providers in North Carolina to determine barriers and facilitators to care of individuals with SCD in the ED setting. ED healthcare providers encounter several challenges as they provide care to SCD individuals. Our qualitative data provided context that helped explain our survey results. We were surprised at the strong endorsement of systems supports, including having adequate knowledge and training (98.2%), administrative support in the ED (98%) and even good nursing staff ratios (80.6%), and not surprised at the high endorsement of overcrowding as a barrier to care described in both surveys (64.86%) and focus groups. There was discordance between the survey responses indicating good nursing staff ratios (80.6%) and comments made during the focus groups about how difficult it was for nurses to administer opioids every 20 minutes. These findings suggest a need to improve ED systems to facilitate care of persons with SCD. Strategies to improve SCD care in the ED include creation of dedicated spaces such as infusion clinics or short-stay units for management of acute SCD complications [10,11].

Approximately 70% of the survey participants reported lack of awareness of the NHLBI recommendations for ED SCD management. However, majority of the interview participants were aware that the guidelines existed but admitted being uniformed on what the guidelines entailed revealing a gap between implementation and actual utilization of the guidelines. In addition to standardized guidelines, participants in the interviews advocated for the use of individualized pain protocols drafted by the patients’ primary care physicians or hematologists. The guidelines and protocols would ensure optimum pain management and facilitate decision making concerning ED or inpatient management of SCD complications. In accordance with previous literature, participants in this study indicated that the use of electronic medical records and individualized protocols facilitated SCD care, particularly if the patient was affiliated with the hospital the ED provider worked in [12–17]. However, access to electronic health records of patients unaffiliated with the hospital proves to be a daunting task in the United States due to interoperability challenges among hospitals using different electronic medical record softwares and administrative requirements set by the Health Insurance Portability and Accountability Act (HIPAA) that need to be met prior to data sharing [18–20].

Stigma was another significant barrier noted in our study. Phrases such as ‘frequent flier’ or ‘drug seeker’ featured prominently in the interviews. The most common barriers related to stigma that were noted in the survey included patient behavior (67.57%), the opioid epidemic (67.57%), concern about addiction (49.55%), stigma around SCD (41.44%), implicit bias (39.64%) and provider attitudes (38.74%). The current opioid epidemic in the United States, which contributed to 70, 237 drug overdose deaths in 2017, impacts SCD stigma [21,22]. Pain is the leading cause of hospital visits in SCD [23]. Other than opioids, very few effective treatments exist for SCD pain management. As a result, SCD patients are often stigmatized as “drug seeking” [23]. Stigma leads to delays in treatment delivery, under-dosing of opioids and poor SCD pain management [23–25]. A proposed strategy to mitigate stigma noted in the interviews was to improve provider knowledge and empathy. Studies exploring stigma in healthcare settings highlight provider training in cultural competency, communication, and the use of self-assessments and patient rating instruments as probable solutions to improve provider knowledge and mitigate stigma towards SCD patients [24,25]. However, to our knowledge, there is no validated measure to assess and address stigma towards SCD patients presenting in the ED. Interventions specifically targeting ED provider attitudes towards these patients are warranted [26].

Finally, a major barrier to care provision in our study noted in both the surveys and interviews was the inability to establish follow-up appointment with a hematologist or a primary care physician. Inability to establish follow-up appointments was attributed to lack of awareness on how to contact outpatient clinics, time constraints and high ED patient volumes. To facilitate contact between the ED and outpatient clinics, Hospital A had an electronic appointment software that enabled ED providers set up follow-up visits with primary care physicians and hematologists. However, majority of the focus group participants from Hospital A reported difficulty with the software or reported time constraints and high patient volumes, which led to poor utilization of the software: this finding has also been noted in previous literature that examined the use of electronic appointment softwares [27,28]. A study on a website referral system by Murnik et al revealed that in spite of proper utilization of appointment software, patient adherence to follow up appointments was still poor indicating the need for more effective methods for establishing linkages to primary care physicians [28]. Interview participants proposed that assistance from social workers or case-managers could be used to establish follow-up appointments, ensure care coordination among various providers, and offer assistance to other patient concerns such as transportation to clinics and psychosocial support. Previous studies demonstrate that effective linkages to outpatient services using case managers leads to reduced utilization of the ED, better retention in care and improved disease outcomes [29,30].

Our study has several limitations. The interviews were conducted in three academic teaching hospitals in a suburban and urban setting in central North Carolina and may not be representative of other hospitals and geographic regions in North Carolina. The interviews were also conducted primarily with physicians and nurses in the ED. Our sampling limits the generalizability of these findings to other healthcare providers with different specializations, though the main focus of our study was the ED. Despite these limitations, we were able to achieve thematic saturation implying the possibility of having missed other factors in the interviews is minimal. Our study also included surveys that were distributed to health facilities all over North Carolina expanding the scope of data.

## Conclusion

The primary barriers to care were overcrowding and concerns about addiction. Suggested solutions to improving care included: the use of the NHLBI guidelines; including individualized pain protocols; use of electronic medical records; and strategies to improve provider knowledge and mitigate stigma. Further research is needed to implement specific strategies to address these issues and assess their effectiveness in improving care to persons with SCD in the ED.

## Supporting information

S1 File Focus group and interview guide. (PDF)

S2 File Survey guide. (PDF)
