# Supplementary material for: Barriers and facilitators to care for individuals with sickle cell disease in central North Carolina: The emergency department providers’ perspective
Source: PLoS One. 2019 May 7;14(5):e0216414. doi: 10.1371/journal.pone.0216414 (PMC6504169; doi:10.1371/journal.pone.0216414)
Supplement: S1 File — (PDF) [file pone.0216414.s001.pdf]

Sickle Cell Disease Implementation Consortium Needs Assessment:
ED Provider Focus Group and Individual Interview Guide

Session Information

Interview date:
Interview team:
Participant ID number:

Participants Information (see demographic form)

INTRODUCTION: ED PROVIDERS

Intro statement for facilitator to read: The purpose of this interview/focus group, is to understand challenges to and resources supporting implementation of the NHLBI recommendations for treatment of SCD, specifically treatment of VOE, into routine practice. This information will help us move forward with strategies to implement the recommendations and improve health for individuals living with SCD.

***Overarching question:* Please describe your practice and the population of individuals with sickle cell disease you see in practice.**

- For example, what are the approximate numbers of patients, age ranges, demographic and clinical characteristics, etc.?
- What is the most common reasons for patient visits?

INTERACTIONS WITH SCD SPECIALISTS AND PCPS

1. ***Overarching question:* Tell me about how you interact with SCD specialists or PCP's related to SCD patients.**

   Probes:

   - Do you have a provider you can refer patients to?
   - Do you have other resources you can refer patients to?
   - Can you refer patients to case management?

OPIOIDS IN THE ED

2. ***Overarching question:* Tell me about your treatment for sickle cell pain?**

   Probes:

   - Tell me about your comfort level prescribing opioids for treatment of VOC in the ED?
   - How do you decide how to treat a patient with sickle cell pain?
   - How do you decide which drugs and routes of treatment to use?

- Is there anything you would change about the care you provide patients during sickle cell pain episodes?
- Are you aware of the NHLBI recommendations for treatment of sickle cell pain?
- What are the barriers to providing good pain management in your ED?
- Are there specific challenges or barriers that adolescents face in your ED?
- What would you need to provide better care to sickle cell patients in your ED?
- Tell me how you advocate for patients right to pain medication.
- Tell me how you ensure opioid safe prescribing practices?
- Tell me about how and when you prescribe opioids at discharge.
- What factors most influences the likelihood you would prescribe opioids at discharge?
