# Supplementary material for: Barriers and facilitators to care for individuals with sickle cell disease in central North Carolina: The emergency department providers’ perspective
Source: PLoS One. 2019 May 7;14(5):e0216414. doi: 10.1371/journal.pone.0216414 (PMC6504169; doi:10.1371/journal.pone.0216414)
Supplement: S2 File — (PDF) [file pone.0216414.s002.pdf]

Subject ID

Sickle Cell Disease Implementation Consortium Needs Assessment: ED Provider Survey

Before we get started with the survey, please tell us whether you primarily provide care through an Emergency Department (ED), and how this survey is being administered.

1. Do you primarily provide care through ED?
   - ❑ Yes
   - ❑ No
2. How is this survey being administered?
   - ❑ Electronically
   - ❑ In-person interview
   - ❑ By phone

A. Emergency Department (ED) Care

1. Does your ED have protocols for treating Sickle Cell pain?
   - ❑ Yes
   - ❑ No
   - ❑ Don't know
   - ❑ Prefer not to respond
2. Does your ED use individualized dosing protocols to treat Sickle Cell pain?
   - ❑ Yes
   - ❑ No
   - ❑ Don't know
   - ❑ Prefer not to respond
3. Are you aware of the NHLBI recommendations for the treatment of Vaso-Occlusive Crisis (VOC)?
   - ❑ Yes
   - ❑ No
   - ❑ Prefer not to respond

B. Please indicate your level of agreement with the following statements regarding taking care of persons with Sickle Cell Disease (SCD) in the ED.

		Strongly disagree	Disagree	Agree	Strongly agree	Don't know	Rather not provide
1.	I have the knowledge to provide care to person with SCD.	❑	❑	❑	❑	❑	❑
2.	I have the training to deliver care to the person with SCD.	❑	❑	❑	❑	❑	❑
3.	I have the administrative support I need to treat patients with SCD.	❑	❑	❑	❑	❑	❑
4.	I have access to medications I need to treat pain in individuals with SCD.	❑	❑	❑	❑	❑	❑
5.	I am able to make a follow-up appointment with a sickle cell specialist following discharge.	❑	❑	❑	❑	❑	❑
6.	I am able to make a follow-up appointment with a primary care provider following discharge.	❑	❑	❑	❑	❑	❑
7.	I am able to refer patients to a case management program upon discharge.	❑	❑	❑	❑	❑	❑
8.	I work in an ED with sufficient nurse staffing to provide good pain management to persons with SCD.	❑	❑	❑	❑	❑	❑
9.	I work in an ED with sufficient physician/provider staffing to provide good pain management to persons with SCD.	❑	❑	❑	❑	❑	❑
10.	Nursing staff ratios allow our ED to provide safe care.	❑	❑	❑	❑	❑	❑
11.	Our nursing staffing allows our ED to provide high-quality care.	❑	❑	❑	❑	❑	❑
12.	A lack of insurance, or being under insured does not affect my ability to provide good care.	❑	❑	❑	❑	❑	❑
13.	The workflow in our ED is conducive to proving high quality care for sickle cell pain crises.	❑	❑	❑	❑	❑	❑

14. Upon discharge from the ED for sickle cell pain, do you prescribe Scheduled-II medications (i.e. opioid analgesics) to patients who request them?

- ❑ Yes
- ❑ No
- ❑ I have never taken care of a SCD patient
- ❑ I have never been asked by an SCD patient for opioid analgesics for their pain management
- ❑ Prefer not to respond

If no, what are the barriers to prescribing Scheduled-II medications (i.e. opioid analgesics) to patients who request them? ____

C. Barrier to care for individuals with SCD

1. Please check all barriers to caring for individuals with sickle cell disease in your ED. *(Please check all that apply.)*

   - ❑ Overcrowding
   - ❑ Implicit bias
   - ❑ Lack of medical equipment (i.e. monitors)
   - ❑ Comfort with level of doses ordered
   - ❑ Opioid epidemic
   - ❑ Lack of care pathway/protocol
   - ❑ Administrative support
   - ❑ Stigma around sickle cell
   - ❑ Social work support
   - ❑ Psychiatric support
   - ❑ Concern about addiction
   - ❑ Provider attitudes
   - ❑ High patient ratios
   - ❑ Patient behavior
   - ❑ Other
   - ❑ Don't know
   - ❑ Prefer not to respond

   If "Other" please specify: ____

D. Demographics Section

2. What is your age?

   - ❑ ___________ Years
   - ❑ Prefer not to provide

3. What is your gender?

   - ❑ Female
   - ❑ Male
   - ❑ Prefer not to provide

4. What ethnicity do you self-identify with?

   - ❑ Non Hispanic or Latino
   - ❑ Hispanic or Latino
   - ❑ Prefer not to provide

5. What race do you self-identify with?

- ❑ American Indian or Alaskan Native
- ❑ Asian
- ❑ Native Hawaiian or Other Pacific Islander
- ❑ Black or African American
- ❑ White
- ❑ Prefer not to provide

6. What is your provider type?

- ❑ Medical Doctor
- ❑ Physician's Assistant
- ❑ Nurse Practitioner
- ❑ Licensed Practical Nurse
- ❑ Registered Nurse
- ❑ Social Worker / Therapist
- ❑ Other
- ❑ Prefer not to provide

If "Other" professional training, please specify: ___________________________

7. How many years have you been in clinical practice?

- ❑ ___________ Years
- ❑ Prefer not to provide

8. What is your practice setting?

- ❑ Rural
- ❑ Urban
- ❑ Suburban
- ❑ Prefer not to provide
